# A multiattribute utility evaluation of different methods for the detection of enteric protozoa causing diarrhea in AIDS patients

**DOI:** 10.1186/1471-2180-10-11

**Published:** 2010-01-15

**Authors:** Lekha Tuli, Deepak K Singh, Anil K Gulati, Shyam Sundar, Tribhuban M Mohapatra

**Affiliations:** 1 Department of Microbiology, Institute of Medical Sciences, Banaras Hindu University, Varanasi, India; 2 Department of Medicine, Institute of Medical Sciences, Banaras Hindu University, Varanasi, India

## Abstract

**Background:**

Enteric protozoa and sporozoa have emerged as important opportunistic parasites and can cause fatal infections in AIDS patients. The line of treatment being different for them necessitates an accurate and prompt identification of these to avoid empirical treatment. In this study which is the first of its kind from India we did a comprehensive evaluation of different techniques, comparing them on the basis of the attributes like yield, cost, time taken, expertise and infrastructure. For the first time combination of Calcoflour White and DAPI, a nuclear stain, were used to identify *Microsporidia *spp. Thus, a diagnostic protocol was devised for rapid, sensitive and cost effective identification of the opportunistic enteric protozoa.

**Results:**

The organisms isolated from the stool samples of the cases (450 HIV patients) were predominantly *Cryptosporidium *spp., *Microsporidia *spp. and *Cyclospora *spp. Interestingly, the control group (200 relatives of the patients who were HIV negative) showed a high incidence (21%) of *Cryptosporidium *spp. We found a significant increase in the sensitivity of microscopy in detecting *Cryptosporidium *spp. and *Cyclospora *spp. after formol ether concentration. Kinyoun's staining was better compared to Modified safranin staining for *Cryptosporidium *spp. identification. Although ELISA had a sensitivity of 93.25% and specificity of 97% for *Cryptosporidium *spp. detection, we ranked Kinyoun's staining better than ELISA because it is not affordable to most of our patients. For detecting *Cyclospora cayetanensis*, autoflourescence was the easiest and most cost effective method followed by Safranin technique. Combination of Calcoflour White stain and DAPI gave good results for the identification of *Microsporidia *spp. We assessed the above techniques and graded the attributes in the following descending order: cost effectiveness, sensitivity, ease of use and interpretation, time taken for the procedure and batch testing.

**Conclusion:**

Thus, we conclude that a combination of minimum three procedures should be carried out for the screening of stool specimens of HIV positive patients. Kinyoun's staining should be made mandatory for every diarrheal stool sample from HIV patients. Also every laboratory should assign its own value to the attributes and apply Multiattribute utility theory or the Analytical hierarchy process to decide the most appropriate methodology.

## Background

The increase in AIDS awareness has lead to extensive studies on opportunistic infections. Coccidia and sporozoa like *Cryptosporidium *spp., *Microsporidia *spp., *Isospora *spp. and *Cyclospora *spp. have emerged as important parasites. Infection with these protozoa usually causes nausea, low grade fever, abdominal cramps, anorexia and watery motions [1]. In immunocompetant people, the illness is generally self limiting.

However, such a heavy loss of electrolytes due to diarrhea can be an important cause of morbidity and mortality in the immunocompromised patients and needs special consideration.

Over the years, detection of these protozoa has been a challenge. Beginning from examination of small or large bowel biopsy material to different staining techniques and their modifications, several methods have been adopted. Many of these techniques are cumbersome and time consuming. Moreover, some protozoa can be missed out by using just one method. Therefore, rapid and sensitive techniques are needed to give an early diagnosis of these protozoal infections as the results can influence therapeutic intervention.

To the best of our knowledge this study is the first of its kind from India in which we did a comprehensive evaluation of different techniques for the identification of the opportunistic enteric protozoa. The study group comprised of patients hailing from rural families of lower economic status [2]. Therefore, this study was designed to compare direct microscopy, modified formol ether concentration, staining methods, fluorescent microscopy and Enzyme Linked Immuno Sorbant Assay (ELISA) on the basis of the following attributes: yield, cost, time taken, expertise and infrastructure.

## Methods

This study was conducted from January 2006 to December 2008 in the Department of Microbiology, IMS, BHU, Varanasi, India. The Institute ethical committee clearance was obtained to conduct the study.

### Study cases

A total of 450 stool samples of known HIV positive patients who complained of diarrhea were collected from the Anti Retroviral Therapy (ART) centre of SS Hospital and Integrated Counseling and Testing Centre (ICTC), IMS, BHU, Varanasi, India. The samples were collected from the patients as and when they reported and they were duly informed about their samples being used for research purpose to which they agreed. Some of these patients were on HAART.

Subjects who were HIV negative and without diarrhea were not included in the study.

### Controls

Family members of the HIV patients coming from the same environmental background who were HIV negative and had diarrhea were chosen as controls. We collected stool samples from 200 such subjects.

### Direct microscopic examination

Stool samples were collected in wide-mouthed disposable containers and processed immediately. If there was a delay in the processing of the samples, they were preserved at 4°C. The samples were divided into three parts. The first part was subjected to direct microscopic examination. With the help of an applicator stick the stool sample was emulsified in a drop of saline on a clean dry slide and in a drop of lugols iodine on another slide. These were covered with cover slips and observed under the microscope at 400× magnification for the detection of ova and cysts.

### Modified formol ether concentration

The second part of the samples was concentrated by Modified formol ether technique [3]. The fecal specimens were mixed with normal saline and passed through double layered gauze cloth into calibrated centrifuge tubes. Saline was added to make the volume to 11 ml to which 2 ml diethyl ether was added and centrifuged at 10,000 rpm for 10 minutes. This procedure was repeated twice after which the supernatant was discarded and the sediment preserved in sterile containers in normal saline. Ether is classed as an extremely flammable reagent requiring storage in suitable flammable-liquid storage cabinets; therefore, we used ethyl acetate as an alternative. Formalin was not used as it leads to a reduction in the fluorescence intensity of stained spores and being a Polymerase Chain Reaction (PCR) inhibitor, it may interfere with the molecular study of the parasites to be conducted later [4]. After concentration the saline and iodine preparations of the samples were microscopically observed as above.

### Staining

The concentrated samples were used for staining. Thin smears from all the samples were prepared on two different slides. The first slide was stained by Modified safranin technique [3]. In this method 3% acid alcohol was used for fixation. Safranin was used for staining and counterstaining was done by Malachite green.

Kinyoun's staining was used to stain the second slide [3]. The smear was fixed with absolute methanol and stained with Kinyoun's carbol fuschin. Destaining was done by 10% alcohol and the smear was counterstained by Malachite green.

At least 200 oil immersion fields of the above smears were examined for the parasites.

### Fluorescence microscopy

A wet mount preparation of the concentrated samples was made and checked for autoflourescence of *Cyclospora cayetanensis *at 200× magnification with a 330 to 380 nm UV filter.

The use of Calcoflour White (Sigma, USA) for fluorescent labeling of *Microsporidia *spores based on the presence of α-chitin in the inner endospore layer of the spore wall was first introduced by Vávra et al [5]. Calcoflour White stain (10 μl) was added to the same amount of concentrated samples taken on clean, dry slides. The working solution was prepared by making 1:10 dilution of the stock (1%) and adding 0.05% Evan's Blue dye. Slides were examined with the help of UV fluorescence microscope at an excitation wavelength of 405 nm.

A modification of the above method was performed by using a fluorescent probe 4, 6-diamidine-2-phenylindole (DAPI). Equal quantities (10 μl) of stool sample and DAPI (Sigma, USA) were put on a slide and left for 5 minutes. Thereafter, 10 μl of Calcoflour White was added and the slides were air dried. The slides were screened with the help of a fluorescence microscope using 435-485 BA filter.

### Antigen detection

The third part of the unconcentrated stool samples was subjected to sandwich ELISA for *Cryptosporidium parvum *antigen detection. The procedure was performed as per the instructions given in the commercially available kit (IVD Research Inc. CA, USA). Briefly, for every test procedure, 100 μl of each, positive and negative control were added to the first two wells, followed by the stool supernatant (100 μl) in the successive test wells to capture the antigen present in the stool. These were incubated for 30 minutes at room temperature (15-25°C) following the addition of 100 μl of anti-*Cryptosporidium *antibody and incubation for 5 minutes to sandwich the antigen. Further, 100 μl of antisecond antibody conjugated to peroxidase enzyme was added and incubated for 5 minutes. All the above steps were followed by decanting the contents after incubation and washing 3 times with the wash buffer. Thereafter, chromogen (tetramethylbenzidine and peroxide) was added, incubated for 5 minutes and the reaction was stopped by adding 100 μl of stop solution in each well. Eventually, the results were read by ELISA reader at 450 nm.

The samples were labeled positive when concordant results were obtained by any two of the above mentioned methods or agreed upon by two observers in a single slide or when found repetitively positive in different slides of the same sample.

While doing the cost calculations for each procedure, material and reagent costs were taken into account. However, we did not include the cost of any equipment like fluorescence microscope, ELISA reader etc. All values were calculated in 2009 Indian Rupees. The sensitivity of each procedure was calculated. Total time taken for a technique included procedure and screening time. A subjective evaluation was done for the parameters like ease of use and interpretation and the ability to process large number of samples at a time (batch testing). The diagnostic procedures were evaluated and ranked on the basis of Multiattribute utility theory and Analytical hierarchy process which identify, characterize, and combine different parameters to evaluate the ranking of the diagnostic tests in any particular health care setting [6,7]. Each procedure was compared by using a linear ranking scale for every attribute (1 was taken for the least preferable characteristic and 6 for the most preferred one). Thereafter, every attribute was prioritized by comparing and assigning its importance over the other as per the laboratory's infrastructure. Subsequently, priority values were multiplied to the ranks given for each attribute for every technique. Finally, a comparison was done after summing up all the obtained figures for each technique.

### Statistical analysis

The statistical analysis was done by Fisher's exact test and Chi-square test using Graphpad software.

## Results

All the 450 stool samples collected from the cases were screened for parasites. *Cryptosporidium *spp. (36.22%) was the organism more often isolated, followed by *Microsporidia *spp. (23.11%), *Cyclospora *spp. (20.44%) and *Isospora belli *(0.44%) in the HIV patients. There were 21.55% cases of mixed infections of which 9.56% cases showed presence of helminths like *Ancylostoma duodenale*, *Hymenolepsis nana *and *Trichuris trichiura *along with the enteric coccidian. The remaining 17.45% were mixed infections of protozoa. The samples taken from the controls showed 21% prevalence of *Cryptosporidium *spp. However, there was a predominance of helminthic infestation with *Ascaris lumbricoides *(22%) leading the list followed by *Ancylostoma duodenale *(20%). The data is shown in the ensuing table (Table 1).

**Table 1:** Parasites isolated from the stool samples of AIDS patients and normal controls

Parasites isolated	HIV positive patients (Cases, no = 450)	HIV negative persons (Control, no = 200)
*Cryptosporidium *spp.	163(36.22%)	42(21%)
*Microsporidia *spp.	104(23.11%)	-
*Cyclospora *spp.	92(20.44%)	3(1.5%)
*Giardia *spp.	40(8.89%)	-
*Entamoeba *spp.	12(2.67%)	4(2%)
*Isospora belli*	2(0.44%)	-
*Ancylostoma duodenale*	25(5.56%)	40(20%)
*Trichuris trichiura*	16(3.56%)	-
*Hymenolepsis nana*	2(0.44%)	6(3%)
*Ascaris lumbricoides*	-	44(22%)
Mixed infections	97(21.55%)	-

The sensitivity of direct microscopy was found to be 63.19% for *Cryptosporidium *spp. and 65.22% for *Cyclospora *spp. whereas; the specificity was 93.03% and 97.21% for *Cryptosporidium *spp. and *Cyclospora *spp. respectively. However, after concentration of the stool samples the sensitivity increased to 74.84% and 78.26% for the two organisms (Table 2).

**Table 2:** Comparison of the Diagnostic Methods for the identification of the enteric protozoa

Organisms	Microscopy		Staining		Fluorescent microscopy			ELISA
		
	Direct	After concentration	Saffranin	Acid Fast	Autoflourescence	Calcoflour White	Calcoflour White + DAPI	
		
*Cryptosporidium *spp.								
Sensitivity	63.19%	74.23%	83.44%	90.79%	-	-	-	92.02%
Specificity	93.03%	95.82%	98.26%	97.91%	-	-	-	96.12%
PPV	83.74%	90.98%	96.45%	96.1%	-	-	-	97.4%
NPV	81.65%	86.75%	91.26%	94.93%	-	-	-	88.39%

*Microsporidia *spp.								

Sensitivity	-	-	-	-	-	95.19%	97.12%	-
Specificity	-	-	-	-	-	97.69%	98.55%	-
PPV	-	-	-	-	-	92.52%	95.28%	-
NPV	-	-	-	-	-	98.54%	99.13%	-

*Cyclospora *spp.								

Sensitivity	65.22%	78.26%	89.13%	85.87%	97.83%	-	-	-
Specificity	97.21%	98.04%	99.16%	98.6%	100%	-	-	-
PPV	85.71%	91.14%	96.47%	94.05%	100%	-	-	-
NPV	91.58%	94.61%	97.26%	96.45%	99.44%	-	-	-

PPV- Positive predictive value, NPV- Negative predictive value

The *Cryptosporidium *oocysts (4-6 μm) took up the Safranin stain and appeared reddish-orange against a green background. However, only a small proportion of the oocysts stained uniformly. On the other hand, *Cyclospora *oocysts (8-10 μm) appeared as uniformly stained red to reddish-orange structures. Safranin staining showed 83.44% sensitivity and 98.26% specificity for detecting *Cryptosporidium *spp. whereas; it was found to be 89.13% sensitive and 99.16% specific for *Cyclospora *spp. identification.

While screening, the technique missed out 27 samples of *Cryptosporidium *spp. and 10 of *Cyclospora *spp. which were found positive by other methods.

On Kinyoun's staining the *Cryptosporidium *oocysts stained as discernable light pink to bright red structures against a green background. It was 90.79% sensitive and 97.91% specific. While variably stained light pink to deep purple oocysts appeared against a blue green background in *Cyclospora *positive samples. In this case the staining was found 85.87% sensitive and 98.6% specific. False negative results were obtained by this method in 15 samples of *Cryptosporidium *spp. and 13 of *Cyclospora *spp.

The presence of *Cyclospora cayetanensis *was confirmed by its neon blue autoflourescence. The technique had a sensitivity of 97.83%. Besides identifying the 82 out of 84 samples positive by the other techniques, it also detected additional 8 samples containing *Cyclospora *oocysts.

**Figure 1:**
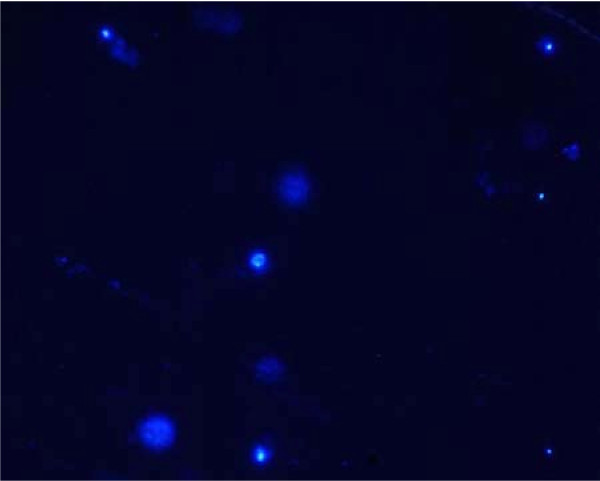
***Microsporidia *spores stained with the combination of Calcoflour White and DAPI**.

*Microsporidia *spp. which were missed by microscopy and staining were revealed as 2-4 μm turquoise white fluorescing structures (Figure 1) on using Calcoflour White technique which was found to be 95.19% sensitive and 97.69% specific. On using the combination of Calcoflour White and DAPI, spores showed an inner bright spot of fluorescence with an increased sensitivity and specificity of 97.12% and 98.55% respectively.

ELISA kit was used for *Cryptosporidium parvum *antigen detection in 376 samples (280 cases and 96 controls). All the 200 samples (160 cases and 40 controls) detected positive by other methods were put to test and an absorbance reading of 0.15 OD units and above indicated presence of *Cryptosporidium *antigen. ELISA gave false negative results in 15 (11 cases and 4 controls) of them. The remaining 176 wells were used for the antigen detection in the microscopically negative samples (120 cases and 56 controls) selected randomly. Of these, 13 samples (8 cases and 5 controls) were read positive for Cryptosporidial antigen. Only 5 (3 cases and 2 controls) of them were confirmed positive for the organism by repetitive staining procedures. Rest of the samples, 5 from cases and 3 from controls were labeled as false positive. The sensitivity and specificity of the assay was 93.25% and 97% respectively.

On applying Multiattribute utility theory and Analytical hierarchy process to the tests employed for detection of the organisms, we rated Acid fast staining almost comparable to ELISA and most appropriate for *Cryptosporidium *spp. diagnosis. For *Microsporidia *spp. both the fluorescent techniques were found equally competent. Autoflourescence detection was found to be the most suitable method for confirming the presence of *Cyclospora *spp. in the samples. (Table 3)

**Table 3:** Ranking of the diagnostic procedures

Techniques	Ranking for the attributes				
	
	Sensitivity	Time taken	Cost	Ease of use and Interpretation	Batch testing
*Cryptosporidium *spp.					

Direct microscopy	1	5	5	1	4
Microscopy after formol ether concentration	2	4	4	2	3
Saffranin	3	2	3	3	2
Acid Fast	4	3	2	4	1
ELISA	5	1	1	5	5

*Microsporidia *spp.					

Calcoflour White	1	2	2	2	2
Calcoflour White + DAPI	2	1	1	1	1

*Cyclospora *spp.					

Direct microscopy	1	4	5	1	4
Microscopy after formol ether concentration	2	3	3	2	3
Saffranin	4	1	2	4	2
Acid Fast	3	2	1	3	1
Autoflourescence	5	5	4	5	5

## Discussion

With the advent of AIDS, parasitic diarrhea has gained a lot of significance. The line of treatment being different for diverse parasites necessitates a definitive diagnosis and study of the etiological agents causing diarrhea, especially when it can be fatal in this vulnerable group of individuals [8].

*Cryptosporidium *spp (36.22%) was the most commonly isolated protozoan in our study was followed by *Microsporidia *spp. (23.11%). As compared to the controls, the observed incidence of these organisms in HIV patients was significantly higher (Fishers exact test, p < 0.0001). In an unpublished report, Samantaray found similar isolation rates in HIV patients from northern India whereas, Ballal from southern part of India showed 9% *Cryptosporidium *spp. and 1.5% *Isospora *spp. Surprisingly, in our study *Isospora belli *oocysts were found in only two samples. This discrepancy in the findings may be attributed to geographical variation. We observed a high prevalence of *Cryptosporidium *spp. (21%) in the control group which comprised of HIV negative family members having diarrhea and coming from similar environmental, social and economic background as that of HIV patients. This interesting finding helped us in tracking the source of infection pointing to water sources contaminated due to continuous shedding of oocysts by HIV positive diarrheal patients and practice of unhygienic toilet habits. Although, the study was conducted to screen for the enteric protozoa but we reported the helminths as and when we came across them.

We found a significant increase in the sensitivity of microscopy in detecting *Cryptosporidium *spp. and *Cyclospora *spp. after formol ether concentration (Chi square test, p < 0.05). As a result concentrated samples were used for further techniques. Mtambo et al reported higher oocysts recovery rates with modified formol ether sedimentation technique than with either sucrose density or zinc sulfate floatation techniques [9].

Similarly, Weber et al reported that sucrose floatation and zinc sulfate floatation yielded lower recovery rates than did the formol ethyl acetate sedimentation method [10].

Waldman et al proposed that ether sedimentation was better than sucrose floatation, as ether extracted lipids from the samples, thus dispersing the oocysts into the aqueous phase [11].

In this study Safranin technique was found to be more sensitive and specific for visualization of *Cyclospora *oocysts compared to *Cryptosporidium *oocysts. Galvan et al also found Safranin technique better for *Cyclospora *oocysts identification [12].

Visvesvara et al found Modified safranin staining to be fast, reliable, easy to perform and superior to Kinyoun's staining for identification of *Cyclospora *spp. [13]. However, Safranin technique required heating and structural details of *Cryptosporidium *oocysts were poorly defined [14]. On the contrary, we found Kinyoun's staining better for *Cryptosporidium *spp. identification compared to Safranin staining. Kehl et al reported Kinyoun staining to be 96% sensitive and 99% specific for *Cryptosporidium *spp. detection [15]. The *Cyclospora *oocysts were variably stained with distorted and wrinkled appearance leading to misdiagnosis. In spite of some individual predilection of the two staining techniques for particular protozoan, they have better diagnostic yields than the unstained smear examination (Fishers exact test, p < 0.05). The staining methods are easy practical, and provisde a stained slide that can be archived. Apart from an advantage of identifying both *Cryptosporidium *spp. and *Cyclospora *spp. the techniques did not show any significant difference between the yields. All the more, both the techniques had kappa indices of 0.85 and 0.90 for *Cryptosporidium *spp. and *Cyclospora *spp. respectively signifying a very good degree of agreement between the two.

Autoflourescence employed for the confirmation of *Cyclospora *spp. was found superior to staining methods (Fishers exact test, p < 0.05). Berlin et al also found a two fold increase in the isolation rates of *Cyclospora *spp. over wet mount [16]. The oocysts of *Cryptosporidium *spp. auto fluoresce so weakly that it is of no value as a diagnostic tool [17]. As per Belli et al UV autoflourescence is consistent with the presence of tyrosine-protein cross links in one or both layers of the oocysts wall [18]. Examination for autoflourescence is a simple, rapid, highly sensitive, inexpensive and easily applicable method to detect *Cyclospora *oocysts in feces. The only requisite being, the availability of a fluorescence microscope.

*Microsporidia *spores displayed variable fluorescence intensities on Calcoflour staining and could be distinguished from the yeast cells by their smaller oval size and absence of budding. Didier et al also stressed upon the advantages of the Calcoflour stain due to its short staining time and high sensitivity both quantitatively and qualitatively [19]. On using DAPI, a nuclear stain which intercalates with the nuclei in combination with Calcoflour White visualization of the spores was better. However, the presence of background 'noise' rendered the technique comparable to Calcoflour White with a kappa index of 0.8954.

ELISA performed to detect *Cryptosporidium *antigen proved to be the most sensitive (93.25%) technique in our hands for indicating the presence of *Cryptosporidium parvum*. Ungar reported the sensitivity and specificity of ELISA as 82.3% and 96.7% respectively in her study [20]. Our study showed higher sensitivity compared to Ungars' because with time the quality of reagents and antibodies being used has undergone a metamorphosis thus improving the assay. With a sensitivity and specificity of 90.9% and 98.7% respectively, Jayalakshmi et al found ELISA to be a simple, reliable and less subjective test which could be very useful in routine diagnosis and for screening a large number of specimens in short time, particularly in large-scale epidemiological surveys [21]. However, Barua et al refute ELISA to be better than staining as the sensitivity of their assay was only 36.4% [22]. The assay may eliminate some of the skill needed in performing complicated staining procedures and recognizing the morphology of the small *Cryptosporidium *oocysts. However, staining holds importance due to its low cost in addition to having a comparable efficacy with the assay.

After the assessment, each attribute was valued as follows; cost effectiveness (0.32), sensitivity (0.30), ease of use and interpretation (0.17), time taken for the procedure (0.13) and batch testing (0.08). We ranked Kinyoun's staining better than ELISA for *Cryptosporidium *spp. detection because ELISA is not affordable to most of our patients hailing from lower economic status. MacPherson et al also gave maximum consideration to cost effectiveness of the tests [23]. Except having lower sensitivity for *Microsporidia *spp. identification Calcoflour White was found to be better in all aspects when compared to the combination of Calcoflour White and DAPI. For *Cyclospora *spp., autoflourescence was the most commendable technique that can be carried out in laboratories equipped with fluorescence microscope and for others Safranin staining could solve the purpose.

## Conclusions

Therefore, we conclude that a combination of minimum three procedures should be carried out for the screening of stool specimens of HIV patients. Besides the direct microscopy, the samples should be subjected to either Kinyoun's staining or Safranin staining and Chromotrope 2R staining or Calcoflour White staining depending on the availability of fluorescent microscope. If not feasible, at least Kinyoun's staining should be made mandatory for every diarrheal stool sample from HIV patients. Since the incidence of *Microsporidia *spp. and *Cyclospora *spp. in the HIV negative patients is negligible, so the screening for these may not be rewarding in this group. Whereas, screening for *Cryptosporidium *spp. is justified in HIV negative family members of the HIV patients due to its high incidence. Also due to difference in infrastructure, expertise and the number of specimens tested every laboratory should assign its own value or utility to the linearly ranked attributes and apply Multiattribute utility theory or the Analytical hierarchy process to decide the most appropriate methodology.

## Competing interests

The authors declare that they have no competing interests.

## Authors' contributions

All the authors read and approved the final manuscript. LT designed the study, performed the experimental work, conceived, drafted and edited the manuscript, DKS helped in drafting the manuscript and statistical analysis, AKG and SS coordinated the study and TMM supervised the study design, coordination of the study and helped to edit the manuscript.
